# Do Metastatic Cells Arise from PD-L1^+^ Cell Niches in Gastric Adenocarcinoma?

**DOI:** 10.3390/ijms27041829

**Published:** 2026-02-14

**Authors:** Erika P. Rendón-Huerta, Ricardo Meléndez-Rendón, At-Sat Aguilar, Luis F. Montano

**Affiliations:** Laboratorio de Inmunobiología, Departamento de. Biología Celular y Tisular, Facultad de Medicina, UNAM, Ciudad de México 04510, Mexico; erendon@unam.mx (E.P.R.-H.); ric_mr@live.com (R.M.-R.); atgape@outlook.com (A.-S.A.)

**Keywords:** gastric adenocarcinoma, PD-L1, metastasis

## Abstract

Gastric cancer (GC), one of the most common malignancies worldwide, is strongly linked to metastasis, significantly worsening prognosis and survival rates. Metastasis initiation relies on epithelial cells undergoing an epithelial–mesenchymal transition and on an abnormal, leaky vasculature. Although the tumor cells involved in the metastatic process have a progression-associated gene signature associated with extracellular matrix organization and the epithelial-to-mesenchymal transition, they must originate from an immune-evasive ecosystem that allows tumors to hinder or evade immune surveillance, either by secreting immunosuppressive chemicals, recruiting regulatory immune cells, or expressing negative stimulatory immune checkpoint molecules such as PD-L1. Although the mechanism underlying the so-called “metastatic cascade” is beginning to emerge, the tumor microenvironment, or niche, in which metastatic cells arise, remains unknown. In this review, we speculate that the epithelial–mesenchymal transition generates PD-L1-expressing cancer stem cells within the primary tumor, which can form tumor niches that serve as sources of metastatic cells within the gastric adenocarcinoma microenvironment. Understanding the regulatory pathways governing metastasis may offer new avenues for developing more effective therapeutic approaches.

## 1. Tumorigenesis

Tumorigenesis is a progressive process in which factors derived from the tumor microenvironment (TME) induce immune tolerance by activating various immune checkpoint molecules [1,2]. One mechanism by which cancer cells favor immune evasion is the expression of inhibitory molecules [3]. The expression of Programmed Death-Ligand 1 (PD-L1), an inhibitory immune checkpoint molecule [4], is regulated by NF-kB downstream of oncogene- and stress-induced pathways and inflammatory cytokines during epithelial–mesenchymal transition (EMT) signaling in gastric adenocarcinoma [5]. This mediating inflammatory suppression suggests that both PD-L1 expression and EMT are part of a tissue remodeling program. The role of epithelial–mesenchymal transition (EMT) in gastric cancer initiation, progression, and immune response resistance has established that EMT transcription factors, including the Snail, Twist, and ZEB families, play pivotal roles in regulating EMT, inflammation, and cancer progression [6,7,8]. Twist is upregulated by PD-L1 but also downregulates TNFα via type I interferons [9], reinforcing its regulatory role in the TME immune system. Nevertheless, the precise molecular mechanisms underlying EMT and the regulation or dysregulation of PD-L1 expression, both of which are relevant processes in the metastatic cascade, remain elusive. There are many therapeutic approaches targeting epithelial plasticity programs [10,11] alone or in combination with PD-1/PD-L1 inhibitors (nivolumab, pembrolizumab) [12]. It has recently been shown that nivolumab plus chemotherapy may benefit patients with advanced gastric cancer [13], but metastatic status influences response to immunotherapy [14]. Hence, understanding the regulatory mechanisms involved in the metastatic cascade is important.

## 2. Immune Checkpoints and Immune Tolerance

Immune tolerance is a state of unresponsiveness to a specific antigen, one of the many mechanisms that cancer uses to evade destructive immunity and maintain tumor persistence in a host with a standard immune system. Central tolerance eliminates self-reactive T and B lymphocytes via clonal deletion, whereas peripheral tolerance inactivates, deletes, or suppresses self-reactive cells that escape central tolerance mechanisms [15,16] because (a) TCR signaling is not followed by co-stimulation [17], (b) repeated antigen stimulation [18], (c) exposure to anti-inflammatory cytokines, or (d) signaling via co-inhibitory receptors such as PD-1 or CTLA [19,20]. Other mechanisms of immune evasion include alterations in MHC-I molecules, abnormal expression of immune checkpoint molecules, increased Treg cells, tumor-associated macrophages, and myeloid-derived suppressor cells [21,22,23].

During tumor progression, the tumor microenvironment is progressively reprogrammed from a tumor-suppressive to a tumor-promoting state [2,24,25] driven by reduced antigen presentation, the secretion of immunosuppressive factors, the accumulation of immunosuppressive cells, dendritic cell hypofunction, and metabolic competition [26,27]. The latter is mainly driven by enhanced glycolysis, which, among other things, regulates immune checkpoint expression [28,29].

The survival of cancer cell clones implies the generation of tolerance as a key feature of the malignant phenotype. Cancer cells produce immunosuppressive cytokines such as TGFβ and IL-10, but they also express PD-L1 downstream of IFN signaling [30] or MYC overexpression or HER2 signaling [31,32]. Overall, it is becoming clear that the heterogeneity and complexity of cellular interactions through which cancers redirect physiological mechanisms to impose a state of immune tolerance are necessary for their survival [33].

## 3. Metastatic Cancer Cell Phenotype

Metastases cause the majority of cancer-related deaths [34]. Still, metastasis is far from an efficient process [35], as it requires specific intrinsic properties of tumor cells, such as a stem-like ability to seed secondary tumors, and extrinsic factors in the microenvironment [36]. In the primary tumor, cancer stem cell clones exhibit individual plasticity and phenotypic dynamics. Numerous mutations and deletions are clearly associated with tumor development and metastatic outcome [37]. These changes have led to the suggestion that metastasis-driven mutations may be tumor type-dependent and that the accumulation of mutations may confer survival advantages to metastatic tumor cells [38]. In pancreatic duct adenocarcinoma, a hypoxic tumor microenvironment activates a metastatic phenotype [39]. The genotype of cells that mediate metastasis encodes secretory or receptor proteins that facilitate tumorigenicity and/or metastagenicity [40], but the stem-like and epithelial–mesenchymal transition features of tumor cells do not necessarily reflect metastatic properties. The analysis of EMT pathways in gastric cancer using a metastatic gene signature confirmed that EMT is the hallmark signature of stage IV gastric cancer progression [41,42] and that the ANKRD6 and ITIH3 genes showed significantly higher expression in patients with metastatic disease; however, not surprisingly, ITIH3 correlated with IL-6/JAK/STAT3 signaling, which is activated by cell-intrinsic PD-L1 [43].

## 4. Immune Checkpoints and Gastric Cancer

In gastric cancer, the tumor microenvironment induces tumor immune tolerance [2], either by transforming mesenchymal stem cells into fibroblasts [44,45], or by mesenchymal stem cells upregulating the Treg cell ratio and increasing PD-L1 expression. Similarly, CD4 T cells can promote PD-L1 upregulation in mesenchymal stem cells [46], thereby promoting EMT and enhancing immune tolerance [47,48].

The predictive value of emerging biomarkers, such as tumor mutation burden, may be influenced by this context. For instance, a high tumor mutation burden could be more effectively leveraged by the immune system in a favorable microenvironment [49], but its benefit might be uncertain in tumors with a predominant EMT phenotype and consequent PD-L1 heterogeneity, for example, in the N-glycosyltransferase STT3-induced cytoplasmic PD-L1 glycosylation through β-catenin, which is critical for EMT.

After recognizing an antigen presented on an HLA molecule, cellular components rearrange to form distinctive immunological synapses upon polarization of the immune cell. PD-1, CTLA-4, and ICOS are key elements of the immunological synapse. PD-1 is a key regulatory molecule of the immunological synapse, expressed on the surfaces of monocytes, dendritic cells, T cells, B cells, and NK cells. When it interacts with its corresponding ligands, PD-L1 or PD-L2, it generates potent inhibitory signals [17]. In gastric cancer, the upregulation of immune checkpoint ligands plays a crucial role in evading immune surveillance [50,51,52,53] as it delivers inhibitory signals to T cells, thereby regulating T cell programmed cell death and maintaining immune tolerance [54,55] and promoting T cell exhaustion [56,57,58].

## 5. PD-L1

The PD-1 ligand, also known as B7-H1, is a 290 aa transmembrane protein with an extracellular N-terminal domain encoded by the CD274 gene on chromosome 9p24.1. It binds to the negative regulators of T cell activation, PD-1 and B7.1 (CD80) [59,60]. PD-L1 is also expressed in antigen-presenting cells, placental cells in an inflammatory microenvironment, and non-hematopoietic cells [61]. In this context, the PD-1/PD-L1 axis maintains the balance between tolerance and autoimmunity by functioning as a negative regulator of self-reactive T cells, preventing co-stimulation. It has been established that tumor-infiltrating T lymphocytes express high levels of PD-1 [62].

**Figure 1:**
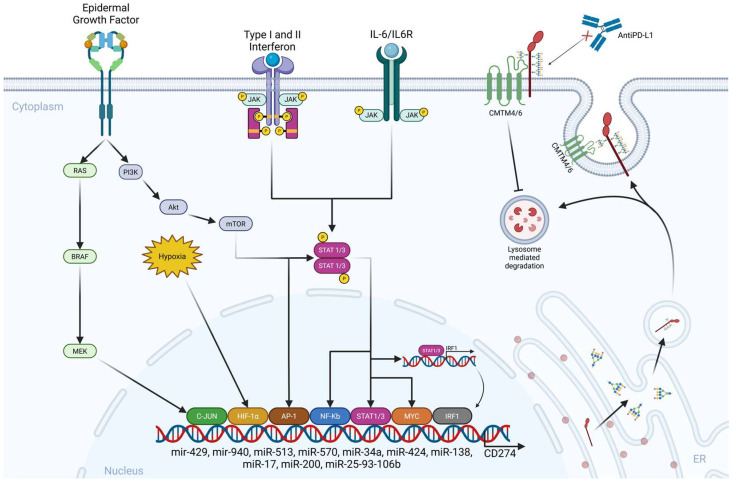
PD-L1 inducers and regulatory mechanisms. Created in BioRender. Meléndez, R. (2026) https://BioRender.com/vu2a0mv.

**Figure 2:**
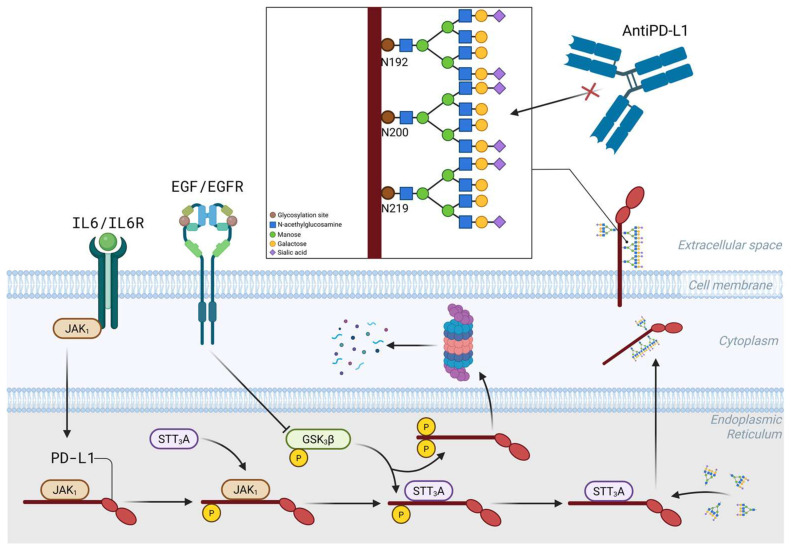
Post-translational modifications of PD-L1. The PD-L1 extracellular domain modifications occur in the lumen of the endoplasmic reticulum. N-glycosylation of PD-L1, mediated by STT3A, requires JAK1-mediated PD-L1 phosphorylation, but N-glycosylation inhibits phosphorylation mediated by Glycogen Synthase 3 beta (GSK3β). STT3A is the human gene encoding the catalytic subunit in the N-olygosaccharyltransferase complex Created in BioRender. Meléndez, R. (2026) https://BioRender.com/8lf9ijs.

PD-L1 is upregulated in a JAK1/JAK2-/STAT1/STAT2/STAT3-dependent manner by type I and II IFNs through IFN regulatory factor 1 [30,63]. Amplification of the PD-L1 gene (CD274) drives PD-L1 expression in only 33% of solid tumors [64,65]. The transcriptional activation of PD-L1 is influenced by transcription factors such as HIF1-a [66,67], Myc, Stat1/3 [68], NF-kB, and AP-1, C-Jun, and IRF1, which are regulated by pathways involving EGF/PI3K/AKT/MTOR, RTK/Ras/Raf/MEK/ERK, IFNγ/JAKs, TLRs/Myd88/Traf6/IKKs, and a lactate-enriched microenvironment [69,70,71,72]. Micro RNAs, especially miR-513, miR-570, miR-34a, miR-424, miR-138, miR-17, miR-200, and the cluster miR-25-93-106b [73,74], have been implicated in regulating PD-L1 expression [75], along with CMTM4/6, a type I transmembrane protein that prevents PD-L1 from lysosome-mediated degradation and maintains its cell surface expression (Figure 1) [76,77,78,79,80,81]. Methylation of the PD-L1 gene inhibits PD-L1 expression [82]. There are two forms of PD-L1: a 45 kDa protein glycosylated at N35, N192, N200, and N219 aa residues that stabilizes PD-L1 on the cancer cell surface and contributes to immune evasion [83], and a 33 kDa non-glycosylated form (Figure 2) [84]. However, the stability of PD-L1 is significantly influenced by post-transcriptional modifications, including glycosylation, promoted by IL6/IL6R and EGF/EGFR signaling, phosphorylation, and ubiquitination [84,85] (Figure 2). A recently described PD-L1 regulatory mechanism in gastric adenocarcinoma is enhanced glycolysis mediated by claudin 9, which facilitates intracellular lactylation of PD-L1 [86]. Post-translational modifications of PD-L1 modulate its biology and impact receptor–ligand affinity and membrane retention [87]. The addition of a sialic acid residue by the ST3 beta-galactoside alpha-2,3-sialyltransferase 4 (ST3GAL4) to the terminal ends of N-glycan chains facilitates PD-L1 interaction with the cell adhesion molecule Sialoadhesin involved in melanoma lymph node metastatic colonization [88]. PD-L1 has two distinct functions: one as a T cell deactivator, allowing tumors to evade the immune system, and the other as a pro-oncogene. Understanding PD-L1 gene amplification and its regulatory mechanisms is necessary to validate its predictive value as a biomarker [89,90]. For instance, it has been established that circular RNA 0000372, acting as a potential oncogene in gastric cancer [91], upregulates the JAK2/STAT3 signaling pathway [92], positively regulating PD-L1 expression; its silencing suppressed cell invasion and immune escape. The likely wrong assumption related to PD-L1 expression is that it is constantly expressed on tumor cells, and it is highly possible that within each tumor microenvironment, prostatic or gastric, there are at least some sensitive cells with low PD-L1 expression and a pre-existing sub-clone of intrinsically resistant cells with a high capacity for PD-L1 upregulation [93,94].

## 6. Epithelial–Mesenchymal Transition

EMT is a cellular process in which cells lose epithelial characteristics (E-cadherin, a tumor suppressor protein) [95] and acquire mesenchymal features (N-cadherin). Type 1 EMT is essential for embryonic development, Type 2 for wound healing and tissue fibrosis, and Type 3 is closely linked to cancer progression [96,97]. Although EMT is considered a reversible and plastic program with different phenotypic stages of progression [98,99,100], it is also a dynamic and pivotal driver of tumorigenesis [7] and has been implicated in tumor development, cancer stemness, and therapeutic resistance [101]. The EMT program is orchestrated by a core group of EMT-inducing transcription factors (EMT-TFs), including the SNAIL family (SNAI1 [102] and SNAI2 [103]); the ZEB family (ZEB1 [104]); and the TWIST family (TWIST1/2) [8]). These EMT-TFs are essential regulators of, among others, therapeutic resistance and tumor immune evasion.

EMT facilitates tumor metastasis via the ZEB family of transcription factors that represses the scaffolding protein WWC1, leading to impaired Hippo signaling and activation of the transcriptional coactivator YAP which induces, among many others, the transcription of PD-L1 [105,106], thus contributing to immune evasion. YAP, a multi-functional regulator in tumor progression and metastasis [107], also promotes EMT through the activation of SNAIL1, SLUG, ZEB1, and TWIST; it is clearly established that the induction of EMT in the gastric primary tumor is followed by enhanced migration [108,109].

## 7. Epithelial–Mesenchymal Transition and PD-L1

Bidirectional regulation between EMT and PD-L1 plays a key role in tumor immune escape [12]. PD-L1 expression is increased by IFNγ, especially in the tumor microenvironment [110]. As already mentioned, N-cadherin, ZEB1, SNAIL1, and Vimentin expression correlates with PD-L1 expression, particularly in epithelial–mesenchymal phenotypes [111,112,113]. A positive correlation between PD-L1 expression and the EMT phenotype has been reported but the contribution of tumor-intrinsic PD-L1 signals to the EMT phenotype is slowly emerging [114,115].

The mechanism by which PD-L1 modulates the EMT process remains ambiguous, but it has been established that EMT upregulates PD-L1 expression via the PI3K/AKT pathway in high-EMT-score cancers [116]. PD-L1 expression is regulated at the transcriptional level by the oncogenic transcription factor Yin Yang (YY1) [117] that is significantly overexpressed in gastric cancer [118] and it is implicated in resistance to immune checkpoint therapies [119] through disruption of the PD-1/PD-L1 axis. YY1 contains an miR-200 binding site [120] and upregulates Snail1 [121]. In fact, two distinct mesenchymal states have been defined by PD-L1 expression and miR-200, which regulate the amount of inducing signal required to undergo EMT [122]. As already mentioned, CMTM proteins mediate many mechanisms driving EMT; for instance, TWIST1 is downregulated by CMTM3 in the gastric cancer cell line AGS [123], thereby inhibiting the metastatic potential of cancer cells [124]. Interestingly, CMTM6, the protein that maintains PD-L1 expression on the cell surface [76], has been linked to the induction of T cell tolerance, cytokine synthesis and secretion, and Treg cell differentiation [125], and is now recognized as a master regulator of PD-L1 expression [126], regardless of the presence or absence of IFNγ stimulation [77]. High expression of CMTM6 has been correlated with peritoneal metastasis in gastric cancer [127]. It is important not to set aside the role of the constitutive oncogenic signaling driven by EMT in the upregulation of PD-L1 [12,128]. EMT and PD-L1 upregulation are well-established drivers of tumor progression, as their bidirectional cross-talk facilitates tumor immune evasion. However, the genuine relationship may be hidden at the single-cell level [129].

## 8. PD-L1 and Cancer Stem Cells

Cancer stem cells (CSCs) are classified into primary CSCs, precancerous stem cells, migratory CSCs, and chemo-radiotherapy-resistant CSCs [130], likely due to metabolic reprogramming that enables them to adapt to their environment and maintain stemness. CSCs acquire a migratory phenotype through EMT [131]. Cancer stem cells, also known as tumor-initiating cells [132], are a distinct subpopulation of tumor cells and key components of the tumor microenvironment, playing roles in immunoregulation and therapy resistance. PD-L1 expression is higher in CSCs than in cancer cells. CSCs possess an efficient redox tolerance system that promotes immunity by selectively inducing PD-L1 expression, which is sometimes mediated by CD44 or by CD44 and CD133 [133,134,135]. For example, CD44+CD24+CD54+EpCAM+ gastric cancer stem cells predict tumor progression and metastasis [136].

CSCs metastasize through a process that involves detachment from the primary tumor, entry into the bloodstream or lymphatic system, survival in circulation, and establishment in distant organs. They accomplish this through EMT mechanisms, which enable them to acquire mesenchymal traits that facilitate detachment. Once in the bloodstream, they survive as they circulate [137,138]. After colonizing a new site, they regain epithelial traits via mesenchymal–epithelial transition mechanisms. These processes require CSCs to maintain their ability to evade immune detection and sustain immune tolerance

Regardless of the CSC markers used to define CSCs across different tumors, most CSCs upregulate immune checkpoint molecules, helping them evade immune surveillance [139]. It is worth noting that environmental factors involved in CSC formation and maintenance, such as hypoxia [140], also contribute to PD-L1 induction. However, it is possible that the overexpression of this crucial molecule, which is essential for allowing tumor cells to evade immune detection and maintain tolerance within the tumor microenvironment, may serve another purpose.

It is well established that PD-1/PD-L1 signaling regulates T cell migration across endothelial cells [141]. PD-L1 is lowly expressed in poorly differentiated and metastatic gastric cancer cells [142]. It is also clear that circulating tumor cells are jettisoned from a single tumor deposit [143,144] composed of tumor cells expressing EMT markers such as CD44 or ICAM-1 [145,146]. PD-L1 expression has been reported on circulating breast cancer cells [147] or on circulating tumor cells isolated from metastatic lung cancer [148], thus enabling evasion of immune cells [149]. Ultimately, understanding the intrinsic properties of circulating tumor cells is an ongoing effort.

## 9. PD-L1 and the Metastatic Cascade

CSCs metastasize through a process known as the metastatic cascade that involves detachment from the primary tumor, entry into the bloodstream or lymphatic system, survival in circulation, and establishment in distant organs. After colonizing a new site, they regain epithelial traits via mesenchymal–epithelial transition mechanisms. These processes require CSCs to maintain their ability to evade immune detection and sustain immune tolerance [137].

**Figure 3:**
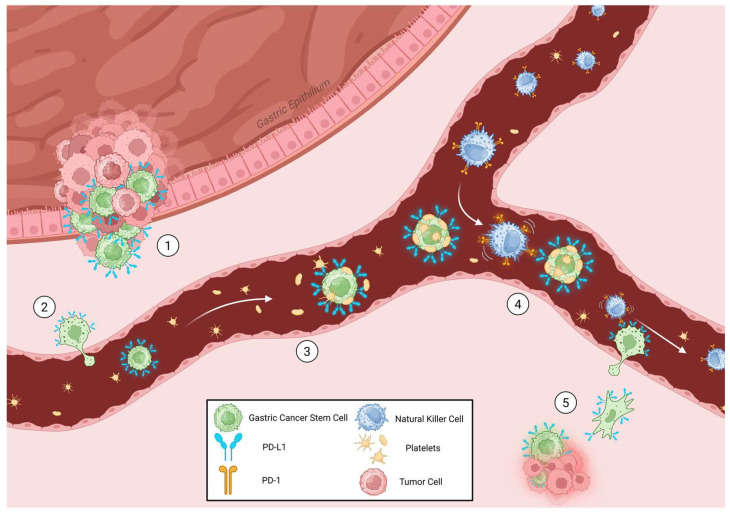
Suggested mechanism of metastatic cascade. (1) PD-L1^+^ gastric CSCs migrate from PD-L1-rich tumoral niche through the extracellular matrix, (2) to the bloodstream by diapedesis. (3) Platelets cover circulating PD-L1^+^ CSCs to avoid innate immune cell signaling through MHC-1 platelet transfer. (4) Subsequently, the CSC/platelet/NK cell complex is created, and PD-1/PD-L1 axis inhibits cytotoxicity activity, (5) allowing the complex to reach the extravasation site, freeing NK cells and inducing secondary tumor site formation. Created in BioRender. Meléndez, R. (2026) https://BioRender.com/8y5jwo3.

The detachment of cancer cells, presumably cancer stem cells, from the primary tumor is associated with EMT and the downregulation of E-cadherin, followed by degradation of the extracellular matrix and intravasation into blood or lymphatic vessels. Once in circulation, cancer stem cells survive and form aggregates with platelets, which can transfer their MHC-I molecules, tricking the immune system [150]. Platelets also act as armor, preventing the most dangerous enemies, NK cells, from spotting and destroying the abnormal cells [151] (Figure 3).

Although the precise origin and phenotype of metastatic gastric cancer cells remain unclear, it is possible that cells that have undergone epithelial–mesenchymal transition [152], thereby replenishing the CSC proportion within the tumor [153], and that express PD-L1 are the source of metastatic cells within tumor niches. Emerging evidence indicates that enhanced PD-L1 expression in tumor-associated neutrophils is the main mechanism suppressing NK cell-mediated antitumor immunity [152] through a STAT3/PD-L1 signaling loop in colon and lung cancer cells [153]. Although there is currently no definitive evidence that our proposal is true, it cannot be ruled out, as PD-L1 expression on the surface of cancer stem cells would protect them from recognition and lytic attack by the tumor environment and circulating NK cells, thereby ensuring the initial steps of the metastatic cascade. NK cells control tumor dissemination, but some circulating tumor cells escape NK cell immunosurveillance by interacting with platelets [154]. Lo et al. showed that NK cells control monoclonal metastasis by eliminating single cancer tumor cells [155]. The relevance of our proposal lies in the idea that metastatic cells arise from PD-L1^+^ cell niches, creating an “immune-privileged” zone that allows these cells to escape NK cell immunosurveillance within the tumor microenvironment and circulation. Targeting PD-L1 might sensitize these cells to conventional treatment, disrupt the protumor microenvironment, and prevent metastasis and recurrence, underscoring the importance of developing new therapeutic strategies, such as the so-called “niche-busting” strategy using CD133-targeting exosomes, to inhibit PD-L1 expression in CSCs.

## Data Availability

The original contributions presented in this study are included in the article. Further inquiries can be directed to the corresponding author.
